# Procalcitonin and lung ultrasonography point-of-care testing to decide on antibiotic prescription in patients with lower respiratory tract infection in primary care: protocol of a pragmatic cluster randomized trial

**DOI:** 10.1186/s12890-019-0898-3

**Published:** 2019-08-06

**Authors:** Loïc Lhopitallier, Andreas Kronenberg, Jean-Yves Meuwly, Isabella Locatelli, Julie Dubois, Joachim Marti, Yolanda Mueller, Nicolas Senn, Valérie D’Acremont, Noémie Boillat-Blanco

**Affiliations:** 1 Infectious Diseases Service, University Hospital of Lausanne, Lausanne, Switzerland; 2 Institute for Infectious Diseases, University of Bern, Bern, Switzerland; 3 Department of Radiology, University Hospital of Lausanne, Lausanne, Switzerland; 4 Department of Outpatient Care and Community Medicine, Unisanté, University of Lausanne, Lausanne, Switzerland; 5 Institute of Social and Preventive Medicine, Unisanté, University of Lausanne, Lausanne, Switzerland; 6 Institute of Family Medicine, Department of Outpatient Care and Community Medicine, Unisanté, University of Lausanne, Lausanne, Switzerland; 7 Swiss Tropical and Public Health Institute, University of Basel, Basel, Switzerland

**Keywords:** Lower respiratory tract infections, Procalcitonin, Lung ultrasound, Antibiotic prescription, General practice, Point of care testing

## Abstract

**Background:**

A minority of patients presenting with lower respiratory tract infection (LRTI) to their general practitioner (GP) have community-acquired pneumonia (CAP) and require antibiotic therapy. Identifying them is challenging, because of overlapping symptomatology and low diagnostic performance of chest X-ray.

Procalcitonin (PCT) can be safely used to decide on antibiotic prescription in patients with LRTI. Lung ultrasound (LUS) is effective in detecting lung consolidation in pneumonia and might compensate for the lack of specificity of PCT.

We hypothesize that combining PCT and LUS, available as point-of care tests (POCT), might reduce antibiotic prescription in LRTIs without impacting patient safety in the primary care setting.

**Methods:**

This is a three-arm pragmatic cluster randomized controlled clinical trial. GPs are randomized either to PCT and LUS-guided antibiotic therapy or to PCT only-guided therapy or to usual care. Consecutive adult patients with an acute cough due to a respiratory infection will be screened and included if they present a clinical pneumonia as defined by European guidelines. Exclusion criteria are previous antibiotics for the current episode, working diagnosis of sinusitis, severe underlying lung disease, severe immunosuppression, hospital admission, pregnancy, inability to provide informed consent and unavailability of the GP. Patients will fill in a 28 day-symptom diary and will be contacted by phone on days 7 and 28. The primary outcome is the proportion of patients prescribed any antibiotic up to day 28. Secondary outcomes include clinical failure by day 7 (death, admission to hospital, absence of amelioration or worsening of relevant symptoms) and by day 28, duration of restricted daily activities, episode duration as defined by symptom score, number of medical visits, number of days with side effects due to antibiotics and a composite outcome combining death, admission to hospital and complications due to LRTI by day 28. An evaluation of the cost-effectiveness and of processes in the clinic using a mixed qualitative and quantitative approach will also be conducted.

**Discussion:**

Our intervention targets only patients with clinically suspected CAP who have a higher pretest probability of definite pneumonia. The intervention will not substitute clinical assessment but completes it by introducing new easy-to-perform tests.

**Trial registration:**

The study was registered on the 19th of June 2017 on the clinicaltrials.gov registry using reference number; NCT03191071.

**Electronic supplementary material:**

The online version of this article (10.1186/s12890-019-0898-3) contains supplementary material, which is available to authorized users.

## Background

As a clear association exists between antibiotic use and resistance rates at community and patient levels, reducing inappropriate use is essential [1, 2]. The highest volume of prescriptions occurs in outpatients presenting with acute respiratory infections (ARIs) [3, 4]. Amongst these, lower respiratory tract infections (LRTIs) are the commonest acute reasons for consulting general practitioners (GPs) [5] LRTIs include acute bronchitis, exacerbation of chronic obstructive pulmonary disease (COPD) and community-acquired pneumonia (CAP). CAP in adults is associated with high morbidity and mortality rates and requires antibiotic treatment [6]. Whereas only 5 to 12% of patients presenting to their GP with LRTIs have CAP, 60% receive an antibiotic prescription [7, 8].

The absence of specific signs and symptoms for CAP makes identifying these patients a challenge [9]; in this context, the presence of a new infiltrate on chest X-ray supports the definite diagnosis [10]. However, chest X-ray has several limitations: it is not always readily available at the GP level [11, 12], exposes patients to radiation and has a limited diagnostic accuracy for CAP (54% sensitivity and 57% specificity, using chest-CT as a gold standard) [13].

Several studies have evaluated the use of host biomarkers to help physicians identify patients with respiratory tract infections potentially requiring antibiotics. Procalcitonin (PCT) and C reactive protein (CRP) are the most extensively studied. PCT is more sensitive than CRP in differentiating bacterial from viral infections in ARIs (respectively 92 and 86%), for a similar specificity (respectively 73 and 70%) [14]. In various settings, including primary care, the use of PCT to guide antibiotic prescription in ARIs reduces antibiotic treatment without affecting outcome [11, 15–17]. Its low specificity in differentiating bacterial from viral aetiologies limits its use in settings with high rates of viral infections, such as primary care. More data are required to confirm the impact of PCT-guided therapy on antibiotic prescription rates among patients with LRTIs in primary care.

Lung ultrasound is effective in detecting lung consolidation and has a 92% sensitivity and 93% specificity using chest-CT as a gold standard for the diagnosis of CAP [13, 18, 19]. GPs or non-physician respiratory therapists can perform and interpret lung ultrasound after a short training course [20, 21]. The maximum exam duration is of 13 min per patient [22]. The high specificity of lung ultrasound to detect lung consolidation could potentially compensate the low specificity of PCT.

We hypothesize that the combination of lung ultrasound and PCT should improve the accuracy of the diagnosis of CAP in primary care. To reduce unnecessary antibiotic prescription without affecting patient safety, we plan to test a novel simple clinical management algorithm (UltraPro) integrating the results **of pro**calcitonin and lung **ultra**sound used as point-of-care tests (POCT).

## Methods/design

### Study objectives

The main objective of the study is to evaluate if the UltraPro algorithm based on procalcitonin and lung ultrasound decreases antibiotic prescription amongst adult patients with LRTIs managed at primary care level in Switzerland compared to the use of PCT only and usual care.

The secondary objectives are to compare the clinical outcome of patients in each arm, to evaluate the acceptability and feasibility of the intervention and to calculate and compare cost-effectiveness between arms.

### Study design and setting

This is a three-arm pragmatic cluster randomized controlled superiority trial conducted in GPs practices.

GPs in 6 cantons in Western and central-Western Switzerland (Bern, Vaud, Neuchâtel, Fribourg, Valais and Jura) were recruited to ensure representation of different cultural areas and antibiotic prescription rates [23]. We chose these regions due to good collaboration between the investigators and a strong network of GPs during previous studies, which is essential for the feasibility of the study [24, 25]. The list of the participating GPs can be found in Additional file 1: Table S1.

**Fig. 1:**
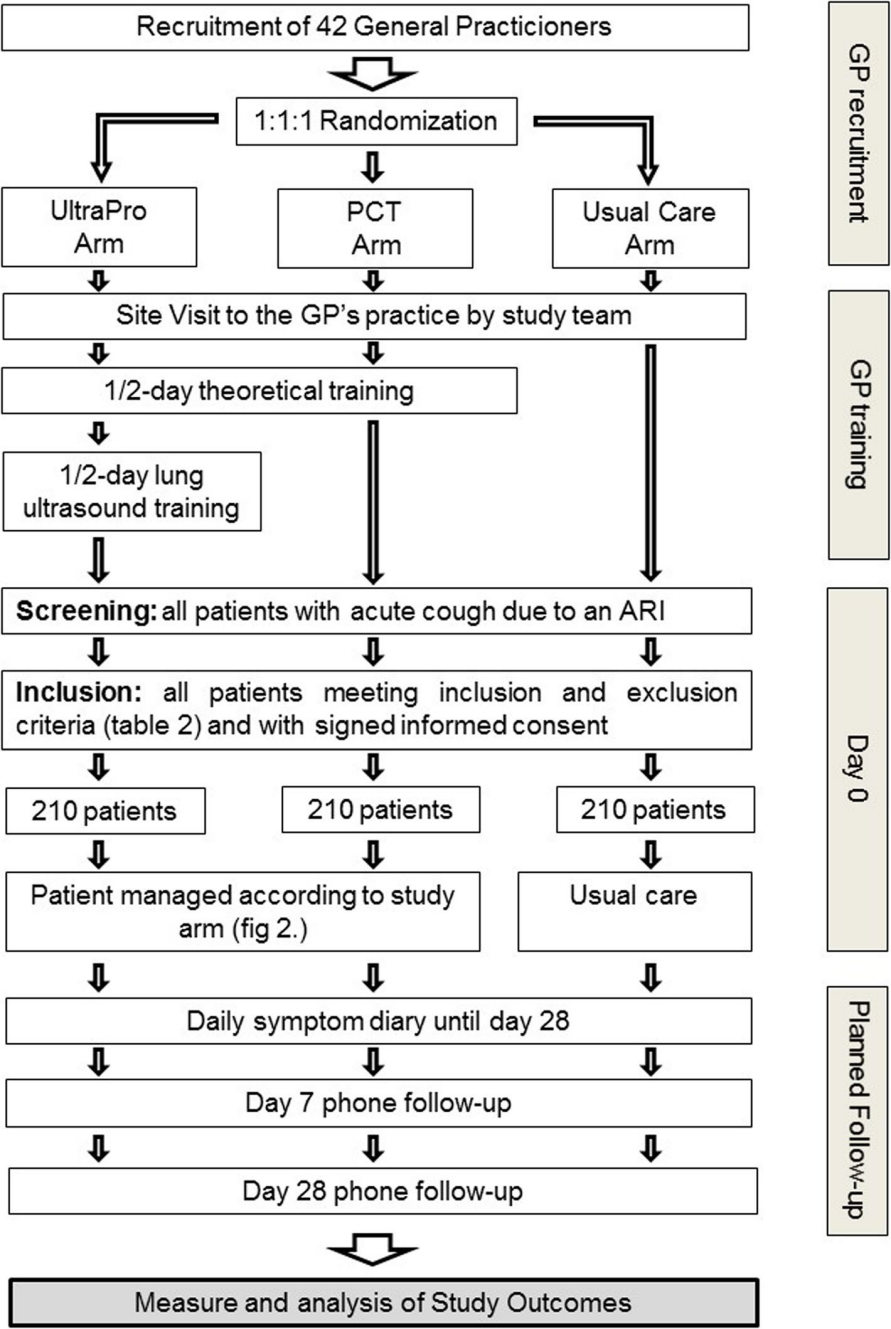
Study design of the randomized intervention study. Abbreviations: PCT: procalcitonin, GPs: general practitioners, ARI: acute respiratory infection, LRTI: lower respiratory tract infection

### Randomization

We will randomly allocate GPs to one of the three arms of the study: UltraPro, PCT only or usual care. Randomization with a 1:1:1 ratio between arms using a computer generated list with variable block sizes will be performed using the REDCap© electronic data capture and management tool [26]. Due to the nature of the intervention, GPs are not blinded.

### Outcomes and hypotheses

The primary outcome is the difference in the proportion of patients prescribed an antibiotic in each arm by day 28. Table 1 summarizes the secondary outcomes.

**Table 1:** Study outcomes

Primary outcome measure	
Proportion of patients prescribed an antibiotic in each arm by day 28	
Secondary outcome measures	
Clinical outcomes	
* By day 7*	
Proportion of patients with clinical failure, defined as: • admission to hospital OR • death OR • absence of amelioration or worsening of relevant symptoms (fever and/or dyspnoea)	
* By day 28*	
Proportion of patients with an adverse outcome, defined as: • admission to hospital OR • death OR complications due to LRTI (persistence of pneumonia, lung abscess, lung effusion, empyema or sepsis)	
Duration of restricted daily activities due to a respiratory tract infection	
Duration of the episode (defined by the total daily symptom score)	
Number of medical visits for the episode of LRTI	
Number of days with side effects related to antibiotics	
Consultation process outcomes	
• Time spent by the patient in the practice, time required for the whole consultation • Patient satisfaction with diagnostic process and consultation outcome • Quality of the ultrasound images and of their interpretation • Provider adhesion, level of trust and perceived usefulness of the algorithm recommendation • Identification of barriers and facilitators to the implementation of UltraPro algorithm in primary care	
Economic outcomes	
Cost / effectiveness ratio	

### Participants

#### General practitioners

GPs known to the investigators for their interest in participating in research and members of local medical societies will be contacted. Study advertising will also be published in GP’s medical journals.

GPs are eligible if they do not use any of the UltraPro POCT tests for the management of their patients with a LRTI in their routine practice. To avoid contamination between arms, only one GP per practice will be included. Each participating GP will be responsible for enrolling 15 patients for a maximum period of 15 months.

#### Patients

GPs will screen for inclusion all adult patients (aged 18 years or older) presenting with a cough due to upper and/or lower respiratory tract infections. They will include every consecutive patient meeting the inclusion and exclusion criteria (Table 2) and providing informed consent (Fig. 1). GPs are responsible for obtaining informed consent.

**Table 2:** Inclusion and exclusion criteria

Inclusion criteria [10]	Exclusion criteria
acute cough (< 21 days) and at least one of the following sign/symptom:	previous prescription of antibiotics for the current episode
• history of fever for more than 4 days • dyspnoea • tachypnoea (> 22 cycles per minute) • abnormal focal finding upon lung auscultation	working diagnosis of acute sinusitis or of a non-infective disorder
	previous episode of chronic obstructive pulmonary disease exacerbation treated with antibiotics during the last 6 months
	known pregnancy
	severe immunodeficiency (untreated HIV infection with CD4 count < 200 cells/mm^3^, solid organ transplant receiver, neutropenia (< 1000 cells/μl), treatment with corticosteroids (dose equivalent to 20 mg prednisone/day for > 28 days)
	decision by the GP to admit the patient
	GP not available for performing study
	patient unable to provide informed consent

### Study intervention

#### Arm 1: UltraPro

**Fig. 2:**
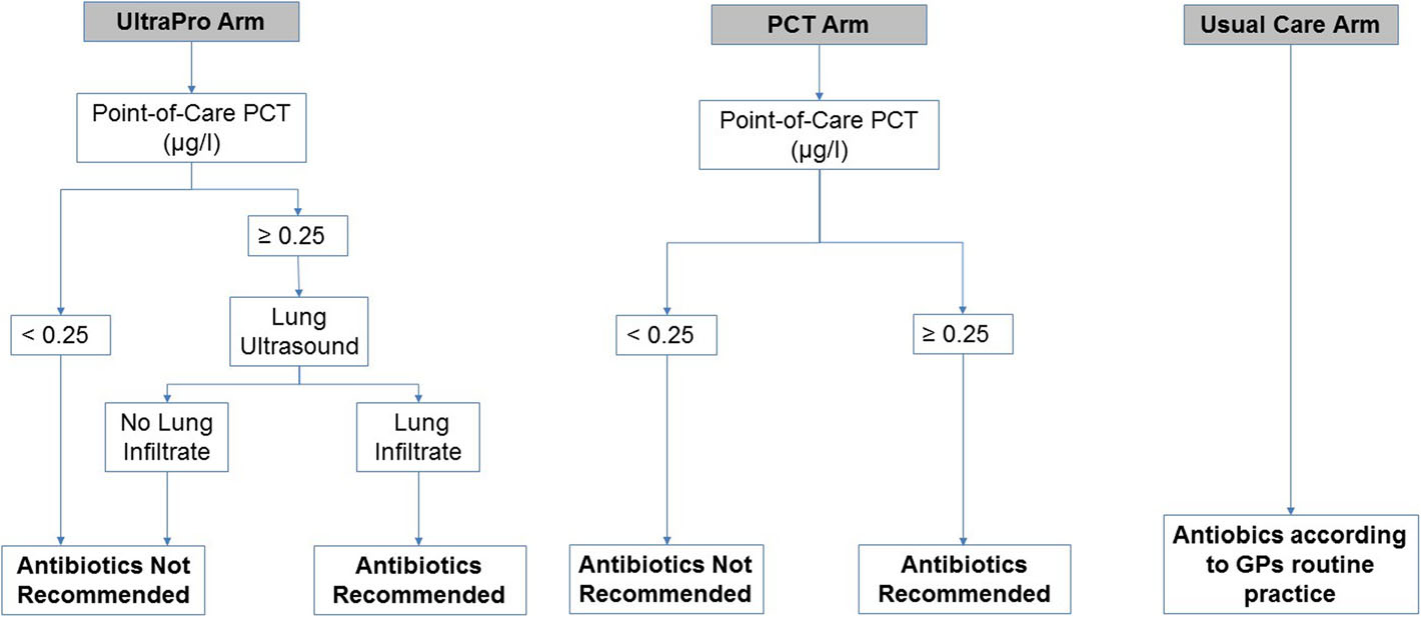
Description of the three arms of the study. Abbreviations: PCT: procalcitonin; GPs: general practitioners

The UltraPro algorithm (Fig. 2) combines the results of a PCT point-of-care test with a lung ultrasound to decide on antibiotic prescription. The medical assistant will measure PCT using the portable Thermo-Fisher© PCT Direct rapid point-of-care test. This immunoassay provides a quantitative PCT result in 20 min using 20 μL of whole blood. This new device has been validated by comparison with the reference method (Elecsys® and Kryptor® B.R.A.H.M.S. PCT assays) with an excellent correlation index (Pearson’s log-r, r^2^ = 0.95) [27]. The measuring range for whole blood samples is 0.22 to 10 μg/l. According to previous studies, a 0.25 μg/L threshold is safe to decide on antibiotic prescription for ARIs at primary care level [11, 17, 28].

In case of an elevated PCT (≥0.25 μg/L), the GP will perform a lung ultrasound with a portable L12–4 convex transducer (Philips© Lumify) to look for the presence of a lung consolidation (Fig. 2). Lung consolidations are defined as sub-pleural echo-poor regions, sub-pleural regions with a tissue-like echotexture or a focal increase of B lines [29]. The procedure will follow international evidence-based recommendations for point-of-care lung ultrasound [29, 30]. We expect the examination to last 10 to 15 min.

Only in the presence of an elevated PCT and a lung consolidation will the algorithm recommend antibiotics (Fig. 2). The GP will choose the antibiotic, its dosage and the duration of treatment. The GP is free to order additional diagnostic tests.

#### Arm 2: PCT only

The medical assistant will measure PCT using the POCT assay described above. Only in the presence of an elevated PCT will the algorithm recommend antibiotics (Fig. 2). The GP will choose the type of antibiotic, its dosage and the duration of treatment. The GP is free to order additional diagnostic tests.

#### Arm 3: usual care

GPs will manage and treat these patients as they usually do (Fig. 2).

### Physician’s training

GPs in the UltraPro and PCT arms will participate in a half-day training session. Topics will include antibiotic resistance, epidemiology of pneumonia in Switzerland, management of CAP in primary care, the use of PCT and lung ultrasound to guide antibiotic prescription, the rationale for the UltraPro algorithm and the procedures of the study.

GPs in the UltraPro arm will participate in an additional lung ultrasonography half-day training session to achieve independent practice and appropriately identify lung consolidation. Topics include ultrasound physics, ultrasound equipment, probe positioning, image recording and interpretation using a phantom simulator (CAE Healthcare©). GPs will take a practical exam at the end of the training session. Three to five months following the start of the study, we will conduct an extra 2 h lung ultrasonography training session with inpatients admitted with CAP.

Before patient recruitment, the study team will visit all GPs at their own practices and train medical assistants in POCT PCT measurement.

GPs in the usual care arm will receive a two-hour training on the rationale and the technical procedures of the study. There will be no training on epidemiology and management of pneumonia.

### Data collection and monitoring

Data collected at inclusion by the GPs include demographics, co-morbidities, symptoms and their respective duration, vital and other clinical signs, laboratory tests and radiologic exams ordered outside the scope the algorithm, PCT result, lung ultrasound interpretation and antibiotic prescription. GPs will record all ultrasound images along with relevant metadata.

Study data will be collected via an electronic case report form (eCRF) and managed using REDCap™ electronic data capture tools hosted at Lausanne University Hospital [26]. In the UltraPro and PCT arms, medical assistants will draw venous blood to perform the PCT POCT measurement.

The study coordinator will perform data monitoring under guidance from the steering committee that is composed of the principal investigator, the co-principal investigators and the co-investigators. The Clinical Trial Unit of the University Hospital of Lausanne will perform the external monitoring of the study.

### Follow-up

A member of the study team (blinded to study arm) will conduct a standardized phone interview of all participants on day 7 and day 28. He will record clinical outcomes (presence or recurrence of LRTIs symptoms), additional medical visits, additional antibiotic prescription, number of days during which activities (work or recreation) were restricted, antibiotic side effects, secondary hospital admission and patient satisfaction.

All participants will fill a validated daily diary until symptom resolution, up to a maximum of 28 days [31]. They will report each day on six items (cough, phlegm, shortness of breath, sleep disturbance, impairment of normal daily activities and feeling unwell) on a Likert scale (1–6). By summing the values, we will obtain a daily composite symptom score for each patient.

In case of follow-up visits, GP’s will manage their patients according to their habitual practice.

### Analyses

#### Sample size calculation

We calculated the sample size to assess an absolute difference in antibiotic prescription of at least 15% between each study arm. The steering committee considered this difference as sufficient to warrant implementation of the intervention at a larger scale. Sixty % of patients are estimated to receive antibiotics with usual care [8]; we expect it to be 45% using PCT only and 30% combining PCT and lung ultrasound.

A study sample of 14 GPs and 15 patients per GP in each arm (210 patients per arm for a total of 630 patients) gives a power of 80% to detect the expected difference in antibiotic prescription with 5% level of significance, when adjusting for clustering at practice level (intracluster coefficient 0.06) [8]. This sample size also guarantees a power of 80% to prove non-inferiority in terms of duration of activities restriction (non-inferiority margin of 1 day, standard deviation of 4 days) as well as in terms of the proportion of patients with adverse outcome by day 28 with an estimated probability in the “usual care” arm of 0.05 (non-inferiority margin of 0.02).

#### Statistical analyses

The population of all patients included, irrespective of follow-up will be used for primary data analysis. For patients who are lost to follow-up we will consider them as having had clinical failure, an adverse outcome and a duration of disease to be equal to the maximum measured in other patients. We will exclude patients whose GPs did not follow the algorithm recommendation and/or who did not complete telephone follow-up of the per-protocol analysis.

The primary analysis will be a logistic regression corrected for variation at GP level (generalized linear mixed effect) to calculate the difference in the proportion of patients prescribed an antibiotic by day 28 as well as the odds ratio of antibiotic prescription between 2 groups.

We will compare secondary outcomes (clinical, consultation process and economic outcomes) between groups using linear mixed effect regression. We will compare episode duration between groups using survival analysis methods.

### Qualitative evaluation

Qualitative assessment will be done by face to face semi-structured interviews with a subset of the participating GPs. Focus groups will also be conducted with a subset of the medical assistants. Interview guides for both the interviews and focus group will focus on needs for training, feasibility of patient’s recruitment and comfort with intervention procedures. We will record all semi-structured interviews and focus groups using standard equipment, transcribed, and coded. Using a content analysis approach, the transcriptions will be firstly analysed to identify key themes and to develop a coding frame. Two different investigators will code independently each transcript and compare them for agreement. The coded data will be analysed and interpreted with regard to the identification of barriers and facilitators to the implementation of interventions processes.

### Economic evaluation

We will conduct a within-trial economic evaluation and complement it with a longer-term economic model. All resources involved in the use of the UltraPro algorithm will be included as intervention costs. These include: 1) human resources: time spent training GPs and medical assistants, additional consultation time and additional time spent by the medical assistant measuring point-of-care procalcitonin, 2) use of health services: referrals to hospital, hospitalizations, unplanned or planned GP consultations, 3) medical supplies and equipment: consumables, investment in devices (PCT reader, ultrasonography transducer).

The eCRF will provide most of the data to measure resource use. Resource use will be valued using the appropriate unit cost, in Swiss Francs (CHF) (e.g. wage rate, tariff, etc.). The within-trial analysis will involve calculating the incremental cost-effectiveness ratio, expressed in CHF per percentage point reduction in antibiotic prescription using the UltraPro algorithm as opposed to PCT only and usual care. In order to incorporate the potential wider benefits of a reduction in antibiotics prescriptions, such as reduced anti-microbial resistance, we will design a long-term economic model.

### Time plan for the study

Patient recruitment will begin in September 2018 and last until February 2020.

## Discussion

This will be the first trial to assess POCT PCT measurement as well as its combination with lung ultrasonography for deciding on antibiotic prescription in patients with LRTIs presenting to primary care. Frequent inappropriate antibiotic prescription in this population is in part due to diagnostic uncertainties surrounding the non-specific clinical presentation of patients with LRTIs and to the low performance of available radiological tools. Physicians need better and easily implemented POCT tools to help them decide on antibiotic prescription in this population.

Our study has several strengths. The chosen randomization level, i.e. GP level, will minimize bias by avoiding contamination between arms that could occur if we randomized at patient level. The screening strategy targeting all patients with cough due to an ARI will allow us to evaluate the proportion of patients with ARIs in whom CAP is suspected and the overall proportion of ARI patients receiving antibiotics. These data will allow us to grasp the potential impact of our intervention from a public health perspective. The inclusion criteria are the clinical criteria for suspecting CAP according to European guidelines. These stringent inclusion criteria guarantee the reproducibility of the study in various settings and avoid using the GP’s subjective clinical diagnosis, as done in previous studies [17]. They also help clinicians in real-life practice target patients who will benefit from additional diagnostic tests after their clinical evaluation. As reflected by our inclusion criteria, our intervention targets only patients with a higher pre-test probability of definite pneumonia. We chose not to include patients with acute bronchitis or asthma exacerbation as it is established that such patients do not require antibiotics [32].

The intervention should not substitute clinical assessment; it completes it by introducing new easy-to-perform tests in case of suspected CAP. Recent studies that did not show an impact of PCT-guided antibiotic prescription in LRTIs included patients, for whom antibiotics were not indicated on clinical grounds alone, thus diluting any potential effect of the intervention [33].

There will be exclusion of a well-defined subset of co-morbid patients (severe underlying lung disease, severe immunosuppression, etc. …) who when presenting with an LRTI should receive antibiotics regardless of clinical presentation, as there are no data showing that they can be safely managed using procalcitonin and lung ultrasound.

As this is a pragmatic trial, the chosen comparator group is “usual care” rather than standard of care. We intend to compare our intervention to real life clinical practice and clinicians in the usual care arm will manage their patient as per their habitual practice. We consider that GPs are aware of existing clinical guidelines and that there are practical and/or pragmatic reasons for not following recommendations.

There will be a PCT only arm. Although PCT is a sensitive test that can safely reduce antibiotic consumption among patients with ARIs [11, 16], more data are needed to confirm its impact on antibiotic prescription rates when compared to routine setting amongst primary care patients with LRTIs. We did not include an additional arm testing lung ultrasound alone as the PCT pre-screening to decide on lung ultrasonography will help save time whilst managing the patients and will be easier to implement at a larger scale in GP practices.

Our study has some limitations. Although we are conducting a pragmatic trial, GP participation is on a voluntary basis, leading to inclusion bias. We expect these motivated and informed GP to have low rates of inadequate antibiotic prescription. This could reduce the effect of our intervention. A reduction in antibiotic prescription rates in this group could then suggest that the real world effect would be greater. We also expect that there will be some deviations from the recommendations of the PCT only and UltraPro algorithms. To reduce the risk of overruling, we present the rationale behind the algorithms to the GPs during the half-day training session and exclude patients who should anyway receive antibiotics when presenting with an LRTI. The possibility of overruling increases the applicability of the findings.

PCT and lung ultrasound are now readily available as POCT making them easy to implement in primary care practices. Use rates of point-of-care ultrasonography in GP practices is variable in Europe [34], it is estimated that around 30% of Swiss GPs have an ultrasound machine at their practice (https://www.doctorfmh.ch/) [35] . This proportion will probably further increase in a near future due to the development of portable and affordable machines together with available short training courses. A pilot study done between December 2017 and April 2018 showed that the implementation of both tools in the proposed setting was feasible. Decentralization of the laboratory analyses and radiologic examination to the GPs practices allows fast and efficient management of patients with LRTIs, and we believe that both PCT and lung ultrasound have a role to play in helping physicians prescribe antibiotics adequately in LRTIs.

## Additional file

Additional file 1: **Table S1.** List of participating GPs. (DOCX 13 kb)

## Data Availability

Not applicable.

## Abbreviations

ARI: Acute respiratory infection
CAP: Community acquired pneumonia
CHF: Swiss francs
COPD: Chronic obstructive pulmonary disease
CRP: C reactive protein
eCRF: Electronic case report form
GP: General practitioner
LRTI: Lower respiratory tract infection
PCT: Procalcitonin
POCT: Point of care testing
