# Supplementary material for: Procalcitonin and lung ultrasonography point-of-care testing to decide on antibiotic prescription in patients with lower respiratory tract infection in primary care: protocol of a pragmatic cluster randomized trial
Source: BMC Pulm Med. 2019 Aug 6;19:143. doi: 10.1186/s12890-019-0898-3 (PMC6683414; doi:10.1186/s12890-019-0898-3)
Supplement: Supplementary file 1 — Table S1. List of participating GPs. (DOCX 13 kb) [file 12890_2019_898_MOESM1_ESM.docx]

| GP’s name | Locality | Canton |
| --- | --- | --- |
| Allain-Pons Béatrice | Lutry | Vaud |
| Anex Frédéric | Echallens | Vaud |
| Antonini-Revaz Sylvie | Lausanne | Vaud |
| Baraschi Constantin | Villars-sur-Ollon | Vaud |
| Barrois Dallières Olivia | Chavannes-près-Renens | Vaud |
| Berdah Daniel | Renens | Vaud |
| Bielinski Rainer | Lausanne | Vaud |
| Blanc Louis | Bern | Bern |
| Brändle Carolina | Renens | Vaud |
| Buff Evelyn | La Sarraz | Vaud |
| Cosandey-Tissot Marjorie | La Chaux-de-Fonds | Neuchâtel |
| Darioli-Pignat Delphine | Moudon | Vaud |
| Dermigny Emilie | Lausanne | Vaud |
| Draeyer Juerg | Wabern | Bern |
| Gennheimer Carl | Visp | Valais |
| Grunder Rolf | Munsingen | Bern |
| Hottinger Michael | Concise | Vaud |
| Huynh Thanh-Liem | Sierre | Valais |
| Jahns Maximilian | Bern | Bern |
| Jaunin-Stalder Nicole | Cugy | Vaud |
| Keravec Erwan | Bulle | Fribourg |
| Klay Michel | Oron | Vaud |
| Lombardo Patrick | Chexbres | Vaud |
| Maire Ponci Céline | Epalinges | Vaud |
| Mayor Vladimir | Colombier | Neuchâtel |
| Minnig Adrian | Bern | Bern |
| Montaru Rozain Corinne | Aubonne | Vaud |
| Morel Abram | Orbe | Vaud |
| Mosimann Madeleine | Bern | Bern |
| Neumann Nicolas | Lausanne | Vaud |
| Pasche Olivier | Thierrens | Vaud |
| Paul Sophie | Gimel | Vaud |
| Persoz Christophe | Neuchâtel | Neuchâtel |
| Rigamonti Véronique | Bern | Bern |
| Ronga Alexandre | Lausanne | Vaud |
| Sagez Julien | Bulle | Fribourg |
| Schwed François | Pully | Vaud |
| Sierro Bard Fabienne | Les Diablerets | Vaud |
| Steiner Anne-Sylvie | Renens | Vaud |
| Vonnez Jean-Luc | Echallens | Vaud |
| Zundel Rolf | Koppingen | Bern |
| Zundel-Maurhofer Doris | Batterkinden | Bern |

**Table S1:** List of participating GPs
